# Overexpression of phospholipid: diacylglycerol acyltransferase in *Brassica napus* results in changes in lipid metabolism and oil accumulation

**DOI:** 10.1042/BCJ20220003

**Published:** 2022-03-31

**Authors:** Stepan Fenyk, Helen K. Woodfield, Trevor B. Romsdahl, Emma J. Wallington, Ruth E. Bates, David A. Fell, Kent D. Chapman, Tony Fawcett, John L. Harwood

**Affiliations:** 1 Department of Biosciences, Durham University, Durham DH1 3LE, U.K.; 2 School of Biosciences, Cardiff University, Cardiff CF10 3AX, U.K.; 3 Department of Biological Sciences, BioDiscovery Institute, University of North Texas, Denton, Texas 76203-5017, U.S.A; 4 NIAB, 93 Lawrence Weaver Road, Cambridge CB3 0LE, U.K.; 5 Department of Biological and Medical Sciences, Oxford Brookes University, Oxford OX3 0BP, U.K.

**Keywords:** crop improvement, MALDI-MS evaluation, metabolic control analysis, oil accumulation, phospholipid:diacylglycerol acyltransferase, triglycerides

## Abstract

The regulation of lipid metabolism in oil seeds is still not fully understood and increasing our knowledge in this regard is of great economic, as well as intellectual, importance. Oilseed rape (*Brassica napus*) is a major global oil crop where increases in triacylglycerol (TAG) accumulation have been achieved by overexpression of relevant biosynthetic enzymes. In this study, we expressed Arabidopsis phospholipid: diacylglycerol acyltransferase (PDAT1), one of the two major TAG-forming plant enzymes in *B. napus* DH12075 to evaluate its effect on lipid metabolism in developing seeds and to estimate its flux control coefficient. Despite several-fold increase in PDAT activity, seeds of three independently generated PDAT transgenic events showed a small but consistent decrease in seed oil content and had altered fatty acid composition of phosphoglycerides and TAG, towards less unsaturation. Mass spectrometry imaging of seed sections confirmed the shift in lipid compositions and indicated that PDAT overexpression altered the distinct heterogeneous distributions of phosphatidylcholine (PC) molecular species. Similar, but less pronounced, changes in TAG molecular species distributions were observed. Our data indicate that PDAT exerts a small, negative, flux control on TAG biosynthesis and could have under-appreciated effects in fine-tuning of *B. napus* seed lipid composition in a tissue-specific manner. This has important implications for efforts to increase oil accumulation in similar crops.

## Introduction

Vegetable oil is a very important agricultural commodity and demand has been rising continuously at 5% each year for the last 50 years [1,2]. This demand is predicted to increase even more as plant oils replace petroleum as a source of renewable chemicals [2]. Since agricultural land is limited, there is an urgent need to boost existing production [3], and one way to do this is to target the biosynthesis of storage oil in seeds [4].

Oilseed rape (*Brassica napus)* is the main oil crop in Northern Europe and Canada and ranks third in the world in terms of total production [5,6]. It is an allotetraploid crop, thought to be a hybrid from unknown varieties of *B. rapa* and *B. oleracea* [7] and can be genetically manipulated relatively easily; indeed, many of the North American plantings are already of transgenic varieties. There have been many studies of oil biosynthesis in oilseed rape [2,6] and its close relation to the model plant *Arabidopsis thaliana* has aided much of this work. In fact, our knowledge of lipid biochemistry in Arabidopsis is already extensive[8].

In oil crops, fatty acids are first synthesised in plastids to yield mainly oleate (18 : 1) and palmitate (16 : 0) (usually in about a 4 : 1 ratio). Fatty acids are then exported to the cytosol where they join the acyl-CoA pool [9–11] and are likely to be bound to acyl-CoA binding proteins (ACBPs, see [12,13]). The acyl-CoAs are then utilised during lipid assembly to provide the three acyl chains of triacylglycerol (TAG). Lipid assembly involves the four steps of the Kennedy pathway [14] as well as an acyl-CoA-independent final step (PDAT, phospholipid: diacylglycerol acyltransferase [15]) for the complete biosynthesis of TAG. Additional reactions allow for modification of the fatty acyl chains (see [10,11]). These additional reactions usually involve phosphatidylcholine as a substrate either for fatty acid desaturation [16] or other modifications [17–19] as well as various acyl hydrolases or transferases [10].

Previous work in our labs. using flux control studies [20] showed that lipid assembly exhibited important control over TAG production in oilseed rape [21–23], confirming the results from previous biochemical experiments [24,25]. In fact, overexpression of DGAT1 (diacylglycerol acyltransferase) shifted control of lipid synthesis significantly [21] and increased oil yields [23]. Moreover, increased lysophosphatidate acyltransferase (LPAAT) activity also augments seed TAG content despite its low intrinsic flux control coefficient [26]. Therefore, in an extension to that work we have generated transgenic lines of *B. napus* cv. DH12075 with further overexpressed enzymes involved in the assembly of TAG.

Phospholipid: diacylglycerol acyltransferase (PDAT) was first reported in plants by Dahlqvist et al. [15] and characterised further by the same group [27]. Since PDAT allows the formation of TAG via an acyl-CoA independent pathway, its relative contribution to TAG formation compared with DGAT has been the subject of considerable study in Arabidopsis (see [11]), where it has been concluded that PDAT is not a major contributor to TAG content in developing seeds [28]. Nevertheless, DGAT1 and PDAT1 have overlapping functions in Arabidopsis TAG biosynthesis and are essential for normal pollen and seed development [29]. In other plants, PDAT may be more important. Thus, in seeds producing highly unsaturated oils such as sunflower, safflower [30], flax [31] or cotton [32], the ability of PDAT to utilise phosphatidylcholine, the site of desaturation reactions, as a direct substrate for TAG formation makes rational sense. Nevertheless, the situation is not entirely straightforward as revealed in a detailed examination of increased expression of DGAT1 or PDAT on seed lipid formation in *Camelina sativa* [33]. PDAT also seems to have an important role for the production of TAG containing uncommon fatty acids such as epoxy or hydroxy fatty acids [34–39]. Moreover, in leaves, PDAT is crucial in diverting fatty acids from membrane lipid synthesis into TAG [40], and its overexpression increased fatty acid synthesis and turnover during TAG accumulation in starchless Arabidopsis mutants [41].

*In vitro* measurements of DGAT and PDAT in oilseed rape [22] and analysis of TAG molecular species formation in developing seeds [4] suggested that DGAT is more important, as might be predicted for a storage oil highly enriched in oleate [1]. This might be similar to the situation in another member of the Brassicaceae family, *Crambe abyssinica* [42], which contains high levels of the monounsaturated fatty acid, erucate, but low amounts of polyunsaturated fatty acids in its seeds [1]. We have previously shown that lipid assembly is relatively important in regulating oil accumulation in *B.napus* [21,22] and to further examine the effect of overexpression of various enzymes for TAG production we have studied PDAT. The results reported here reveal that PDAT overexpression produced effects both on quantitative as well as qualitative aspects of lipid metabolism.

## Experimental

### Plant materials and growth

*Brassica napus* (oilseed rape) cv. DH12075 seeds were obtained from Agriculture and Agri-Food Canada, Saskatoon, Saskatchewan, Canada. Seeds were germinated on wet tissue paper, planted in Levington M2 compost with 5 g/l slow-release fertiliser (Levington, Surrey, U.K.) and grown at 20°C (16 h) day and 15°C night. Flowers were tagged on opening and harvested 27 days after flowering (DAF) when the seeds were at stage III of development [43]. Previous studies [44] had shown this to be mid-way in the period of rapid oil accumulation.

### PDAT1 construct

The *Arabidopsis thaliana* PDAT1 sequence, At5g13640, was synthesised by Genscript, U.S.A. and cloned into pEntr1A-Napin-NosT between the *Brassica napus* Napin promoter and the *Agrobacterium tumefaciens* Nos terminator sequences via *Nde*I and *Eco*RI sites. The Napin promoter-PDAT cassette was then transferred by Gateway LR Clonase II recombination into binary destination vector pRMH009, which contained the *nptII* gene expressed from the Nos promoter, for selection of transformed material in tissue culture (Supplementary Figure S1). The resulting plasmid, pEW227-PDAT1, was verified by restriction mapping and sequencing the insert.

### Transformation methodology

Oilseed rape transformation was carried out as previously described [45] with *Agrobacterium tumefaciens* Agl1 pEW227-PDAT1 transformation of DH12075 cotyledonary petioles. Transformed shoots were regenerated in the presence of 15 mg l^−1^ kanamycin. Rooted shoots were transferred to Jiffy-7 pellets (Jiffy Products, Kristiansand, Norway), acclimatised to growth chamber conditions and transferred to 12 cm pots containing Levington M2 compost with 5 g/l slow-release fertiliser as above. T_0_ plants were self-fertilised and T_1_ seed harvested from all lines.

### Genomic DNA analysis and selection of lines

DNA was isolated from T_0_ plants as previously described [45] and plants were verified as transformed by PCR presence of the gene of interest and absence of the *aphIII* gene, present on the binary vector backbone. T-DNA copy number estimation of the *nptII* gene was determined by quantitative PCR using the ΔΔCt method and the number of T-DNA integration loci were determined by Southern hybridisation as previously described [26]. T_0_ plants with one or two sites of integration and one or two copies of the T-DNA were prioritised for growth of T_1_ plants and seed multiplication for further study. T_1_ plants homozygous for the transgene and *nptII* gene were identified by quantitative copy number PCR. Null-segregant (azygous) T_1_ plants were also identified as controls. Segregation ratios were compared with expected Mendelian inheritance patterns.

### Characterisation of seed lipids

Seed TAG and phospholipids were extracted and analysed as previously described [26]. Individual TAG species were separated using a Waters ACQUITY UPC^2^ system (Waters, Milford, U.S.A.) on a UPC^2^ HSS C18 SB column (3.0 mm × 150 mm column i.d. 1.7 µm) and TAG peaks identified using UPC^2^ photodiode array detector and the exact mass spectra collected by single quadrupole MS [46].

### Radiolabelling of developing embryos

Siliques were harvested at 27DAF and seeds selected for uniformity of appearance. Dissected embryos were pooled and incubations carried out with six replicates, each containing five embryos. Incubations were in 0.1 M potassium phosphate buffer (pH 7.0), 1 M sorbitol containing 1 µCi [U-^14^C]-glycerol (PerkinElmer, Massachusetts, U.S.A.) with gentle shaking for 6 h at 20°C. After incubation, seeds were washed (X3) with unlabelled buffer and heated in isopropanol for 30 min at 70°C to inactivate endogenous lipases. Extraction and separation of individual lipid classes by TLC was as previously described [26].

For manipulation experiments, embryos were pre-incubated with 1 mM oleic acid (Sigma–Aldrich) dissolved in 1 mM tetramethylammonium hydroxide in 0.1 M sorbitol for 2 h with gentle shaking. Control samples were treated as above without oleate. Incubations with 100 µM diazepam (Sigma–Aldrich) contained the inhibitor for the whole 6 h incubation.

### Sample preparation for MALDI-MSI

Dry seeds were embedded in 10% gelatin and then frozen for 16 h at −80°C. Frozen embedded seeds were transferred to −20°C where they were equilibrated for 3 days prior to cryosectioning. Sections of 30 µm thickness were taken using a Leica cryo-microtome (CM1950, Leica Biosystems) and then transferred to Superfrost Plus microscope slides (Fisher Scientific). Sections were then lyophilised for 3 h prior to application of the MALDI-MS matrix 2,5-dihydroxybenzoic acid via sublimation, modified as previously described [47].

### MALDI-MSI of tissue sections and data analysis

Tissue sections were imaged using a MALDI-LTQ-Orbitrap mass spectrometer (ThermoScientific) with the following set parameters: 12 µJ laser energy, 10 laser shots, 40 µm raster step size, collected over an *m/z* range of 700 to 1000 with the Orbitrap mass analyser set to a resolution of 60 000. Data were analysed using the MS imaging software Metabolite Imager [48] and MSiReader [49]. PC molecular species were detected as [M + H]^+^ and [M + K]^+^, and were imaged as the sum of the two adduct intensities for each molecular species identified. TAG molecular species were detected as [M + K]^+^ adducts. Both lipid classes were imaged with a mass tolerance of 10 ppm. MS images were presented as mol% on a colour scale from yellow (low) to blue (high).

The average mol% of PC and TAG molecular species for specific tissues were calculated by summing the ion intensity for each of the individual molecular species for each pixel within an outlined tissue area (embryonic axis, inner cotyledons, outer cotyledons), where each pixel represents a laser spot, and then normalising to the total summed ion count for the lipid class (either PC or TAG). Three replicate MS images taken from sections of different seeds were analysed in this way, with the average and standard deviation plotted in bar charts for each genotype.

### Enzyme assays

Microsomes were prepared from 27DAF embryos according to Berneth and Frentzen [50]. The pellet was re-suspended in 50 mM Tris–HCl buffer (pH 7.6) containing 5 mM dithiothreitol and 20% glycerol and stored at −80°C. Protein was estimated using Bio-Rad bovine serum albumin Protein Assay kit according to the manufacturer's instructions.

PDAT assays were carried out according to Banas et al. [30]. Microsomal samples (20–40 µg protein) were lyophilised overnight. 5 nmol DAG and 5 nmol of *sn*-1-oleoyl-*sn*-2-[1-^14^C]oleoyl-glycero-3-phosphocholine (ScanBiRes, Alnarp, Sweden) were dissolved in benzene and applied to the lyophilised microsomes. The incubation buffer, pre-incubation and incubation conditions were as described [30]. After 90 min at 30°C (shaking at 1250 r.p.m.), TAG carrier was added and lipids were extracted [51]. Lipids were separated on TLC plates using hexane/diethyl ether/acetic acid (70/30/1, by vol) and silica gel G plates and quantified using a Typhoon 9400 Imager (GE Amersham Molecular Dynamics).

### Metabolic control analysis

A quantitative estimate of the degree of influence of PDAT on the rate of deposition of TAG in the seed was made in terms of its flux control coefficient, $\;C_{E}^{J}$, which represents the percentage change in flux *J* to TAG for a 1% change in the enzymic activity, *E*, of PDAT [20]. A more precise definition is:

1 $$C_{E}^{J}=\frac{E}{J}\cdot \frac{\partial J}{\partial E}=\frac{\partial \ln J}{\partial \ln E}$$

The methodology used to determine the flux changes was that developed [52] and justified for the estimation of the flux control coefficient of LPAAT on TAG deposition in oilseed rape in our earlier study of the overexpression of that enzyme [26]. Briefly, this relies on the observation that the time course of TAG accumulation follows exponential growth kinetics, so that the rate constant can be determined from the weight of TAG at the beginning and end of the exponential phase. More specifically, the relative change in the rate constant between a set of control (non-segregant) plants and a set of overexpressors of a given genotype depends on the logarithms of their respective mean final TAG weights per seed.

Since the calculations depend on the TAG accumulated in the seeds during their development on the plant, the resulting flux control coefficients represent an *in planta* estimate where the system under consideration includes all metabolism and processes between fixation of CO_2_ and formation of the TAG droplets.

In the case of the LPAAT experiments, the relative change in the TAG flux could not be used to directly compute the flux control coefficient because it is defined for small changes in enzyme activity about the wild-type level, whereas expression of additional gene copies for the enzyme results in several-fold increases in enzyme activity. Typically, substantial changes in enzyme activity result in a hyperbolic relationship between flux and activity as the flux control coefficient declines, as indeed we observed in our LPAAT studies [26]. In such a case, the calculation of the flux control coefficient in the wild-type plants has to be corrected by use of a formula for large fold changes in enzyme activity. In the experiments reported here, although several-fold changes in PDAT activity were achieved, the flux changes were relatively small and the conditions for application of the large change formula were not met.

Instead, as Eqn. (1) states that the flux control coefficient is the slope of the relationship between the log of the flux against the log of the activity, the flux and PDAT activity measurements for the three lines of overexpressors and their non-transgenic, segregant controls were so plotted. The slope of the straight line fitted to the data points was taken as the *in planta* flux control coefficient of PDAT on TAG accumulation, and the standard error of the flux control coefficient was given by the standard error of the slope.

A second method analysed the three pairs of non-segregant (0) and overexpressor (1) data separately using the finite difference approximation to Eqn. (1):

2 $$C_{E}^{J}\approx \frac{\left(\text{ln}J_{1}-\text{ln}J_{0}\right)}{\left(\text{ln}E_{1}-\text{ln}E_{0}\right)}$$

As previously [26], the uncertainty in each of these values was estimated from a thousand *in silico* experiments where the measurements underlying the evaluation of Eqn. (2) (the seed TAG weights and the PDAT activities) were simulated by randomly drawing them from normal distributions with the means and standard errors of the experimental values reported in Supplementary Table S1.

## Results

### Production and expression of PDAT overexpressing lines

The construct used to produce PDAT overexpressing lines of oilseed rape was derived from the Arabidopsis PDAT1 gene. Its details are shown in Supplementary Figure S1.

Twenty-four independent T_0_ transgenic plants were regenerated from one transformation experiment, and confirmed as transformed by PCR amplification of the PDAT gene of interest plus *nptII* (T-DNA) copy number determination. A transformation efficiency of 9.3% was achieved, calculated as the number of cotyledonary petioles which regenerated a transgenic plant. The number of T-DNA integration loci were determined by Southern blotting using the *nptII* gene as the probe (Supplementary Figure S2(b)). Three T_0_ plants containing a single copy (line 24), or two copies (lines 2 and 33) of the T-DNA insertion, and which were PCR negative for the *aphIII* gene, were selected for T_1_ seed germination to identify homozygous and null segregated (NS) plants for growth to maturity, seed multiplication and detailed characterisation.

Following self-fertilisation, segregation of line 33 enabled identification of plants with single T-DNA inserts at the two different loci. These are designated 33A and 33B, whereas plants which contain the transgene at both sites are designated 33AB.

### Analysis of seed TAG content and PDAT activity

**Figure 1.**
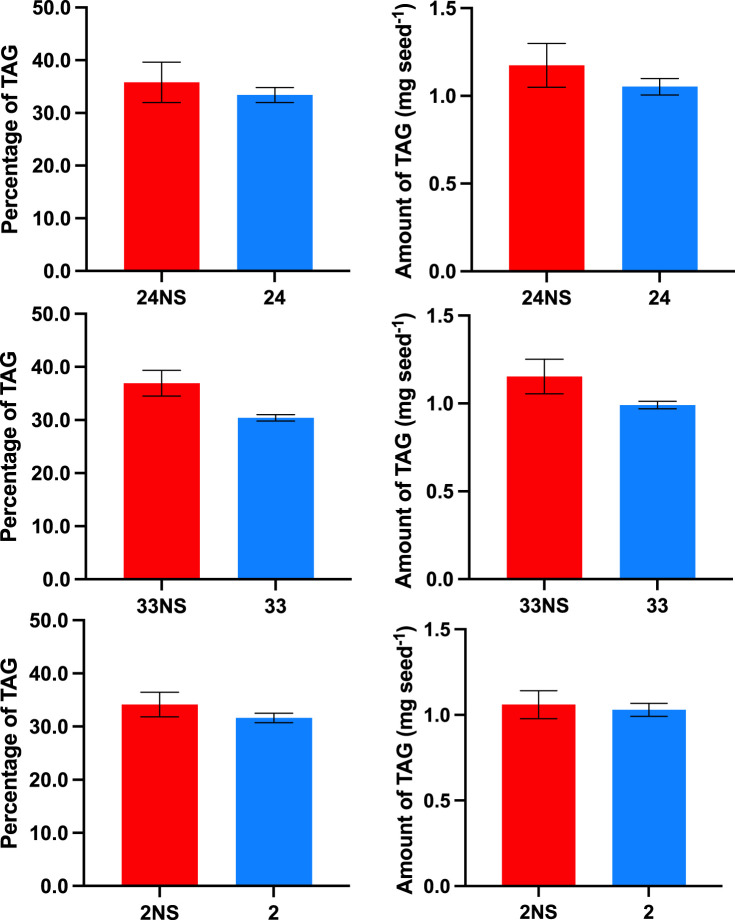
Amount of triacylglycerol accumulated in mature seeds from three PDAT overexpressing lines of *Brassica napus*. Plants were grown as described in Experimental. Data show means ± SE (24NS, *n* = 3; 24, *n* = 18; 33NS, *n* = 6; 33, *n* = 42; 2NS, *n* = 6; 2, *n* = 18) for % dry weight and as mg/seed. NS = null segregant control.

TAG content of seeds from three separate T_1_ lines of PDAT overexpressors was measured and the data are shown in Figure 1. Whether TAG was expressed as % dry weight or as mg per seed, all three lines showed a decrease in TAG, but the decrease was only statistically significant for line 33 at the 95% confidence level, when compared with corresponding null-segregant (NS) values. Results from individual plants of the three lines are shown in Supplementary Figure S3. The mean values for % dry weight TAG in seeds from overexpressing plants were: 34.2 ± 1.6% (SE; NS = 38.7 ± 3.8%) for line 24; 30.4 ± 0.6% (NS = 36.9 ± 2.4%) for line 33; and 31.5 ± 3.61.1% (NS = 34.2 ± 2.3%) for line 2.

**Figure 2.**
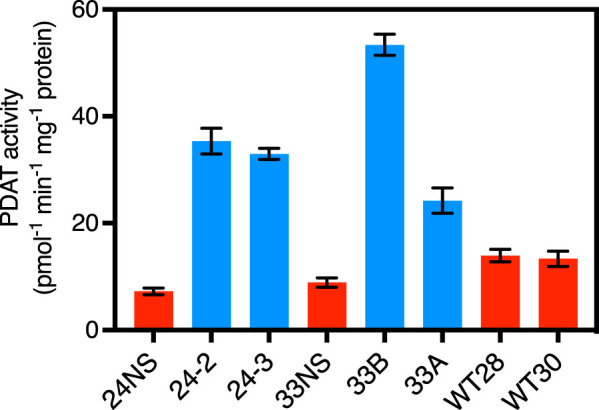
PDAT activity in microsomal fractions of 27 DAF embryos of overexpressing or null segregant lines and wild-type controls of *Brassica napus*. Data expressed as means ± SE (*n* = 3). NS = null segregant control (blue = transgenic overexpressors, red = null segregant or wild-type). WT28 and WT30 are biological replicates.

To measure the PDAT activity in overexpressing lines and their corresponding null segregant controls, we assayed microsomal fractions by the method of [30]. All overexpressing lines showed substantial increases in activity compared either to their null segregant controls or to two independent wild-type samples (Figure 2). The measured activity in wild-type extracts was higher than in non-segregant plants, confirming the latter as the most appropriate controls.

### Flux measurements in developing seeds

**Figure 3.**
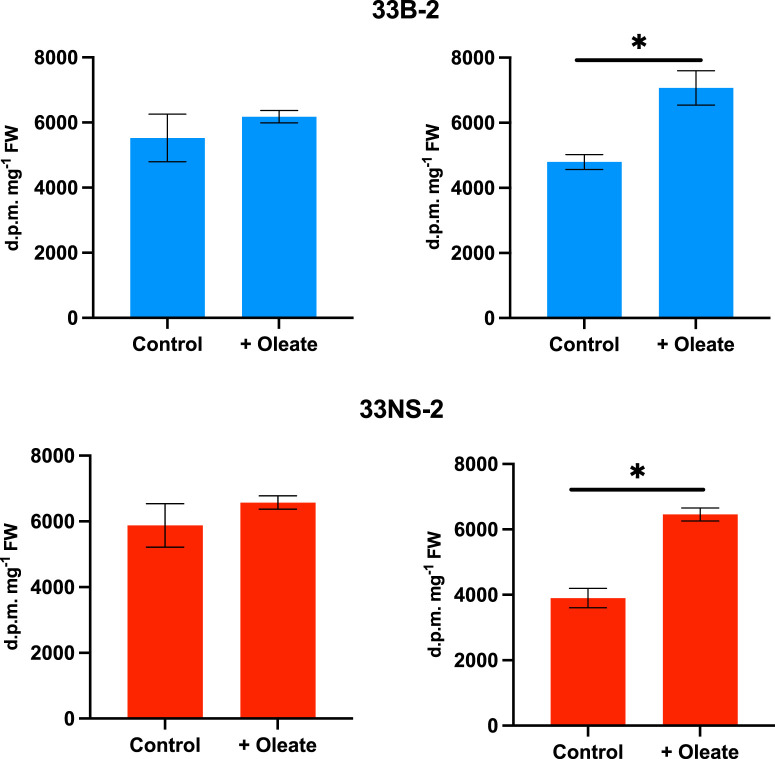
Exogenous oleate increases total lipid labelling from [U-^14^C}glycerol in both a PDAT overexpressor (33B-2) and null segregant (33NS-2) control. Embryos from 27DAF seeds were used and results show means ± SE (*n* = 6 biological replicates). Two separate experiments are shown using different plants, * shows statistically significant changes (*P* < 0.05). Distribution of radiolabel between lipid classes is shown in Supplementary Figure S4.

Reactions involved in the production of acyl lipids can be followed using [U-^14^C]glycerol as a precursor [11,53–55]. In previous flux control experiments with *B. napus* cv. Westar, we showed that exogenous oleate would increase lipid synthesis while diazepam would reduce it (see [22] for background information). We tested 1 mM exogenous oleate in developing (27DAF) embryos from cv. DH12075 and found an increase in the incorporation of radioactivity into embryo glycerolipids in both null segregant controls (33NS) and a related overexpressing line (33B) (Figure 3).

**Figure 4.**
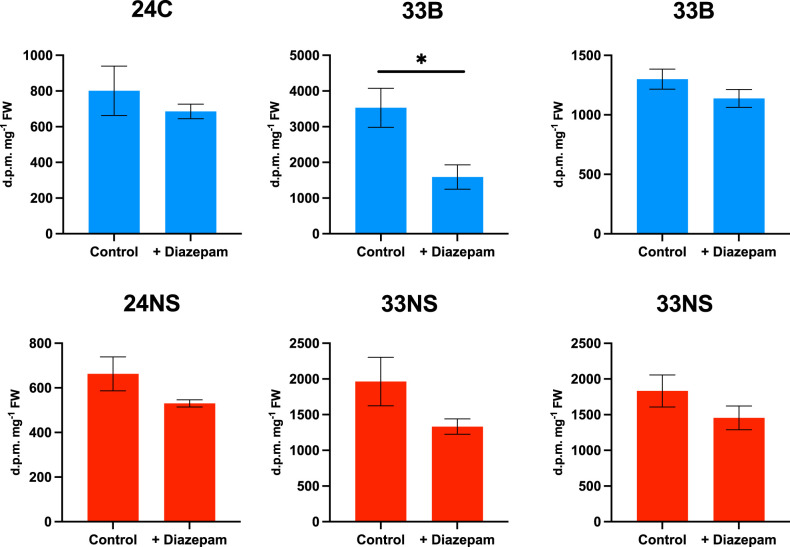
Diazepam reduces lipid labelling from [U-^14^C]glycerol in both PDAT overexpressors and null segregant (NS) controls. Embryos from 27DAF seeds were used and results show means ± SE (*n* = 6 biological replicates). Two separate experiments for line 33B are shown using different plants, * shows statistically significant changes (*P* < 0.05). Distribution of radiolabel between lipid classes is shown in Supplementary Figure S5.

When diazepam was included in the incubations, it inhibited incorporation of radioactivity in both PDAT overexpressors and their NS controls (Figure 4). The magnitude of effects of both oleate and diazepam in the control incubations was similar to data reported before for *B. napus* cv Westar [22].

Following incubations with [^14^C]glycerol, we examined the distribution of radioactivity between lipid classes to see whether addition of either oleate or diazepam altered this. Data are shown in Supplementary Figure S4 for oleate and in Supplementary Figure S5 for diazepam. After oleate addition there were decreases in the % radioactivity in polar lipids and increases in TAG in both NS control and transgenic embryos, though these were not always statistically significant. In contrast, while a decrease in the % radioactivity in DAG was observed in both transgenic lines, an increase in DAG was found with oleate addition in the NS controls (Supplementary Figure S4). When the polar lipids were separated, phosphatidylcholine (PC) and phosphatidylglycerol (PG) were the main labelled lipid classes. In general, oleate addition increased the relative labelling of PC but reduced that of other polar lipids. There were no obvious differences in the % labelling of polar lipids between NS controls and PDAT overexpressing lines (Supplementary Figure S4).

For diazepam addition, where there were also significant changes, the inhibitor increased the % labelling of polar lipids at the expense of DAG. Within the polar lipids, the two main labelled fractions (PC and PG) showed reciprocal alterations. While PG labelling was increased by diazepam, that of PC was decreased (Supplementary Figure S5). This contrasted to the effect of exogenous oleate, which also increased overall lipid labelling as opposed to diazepam that decreased it (Figures 3 and 4). Taken together, these radiolabelling data indicated that overexpression of PDAT had little effect on the flux of carbon through polar lipid biosynthesis and TAG assembly in developing *B. napus* embryos.

### Alterations to endogenous lipids

**Figure 5.**
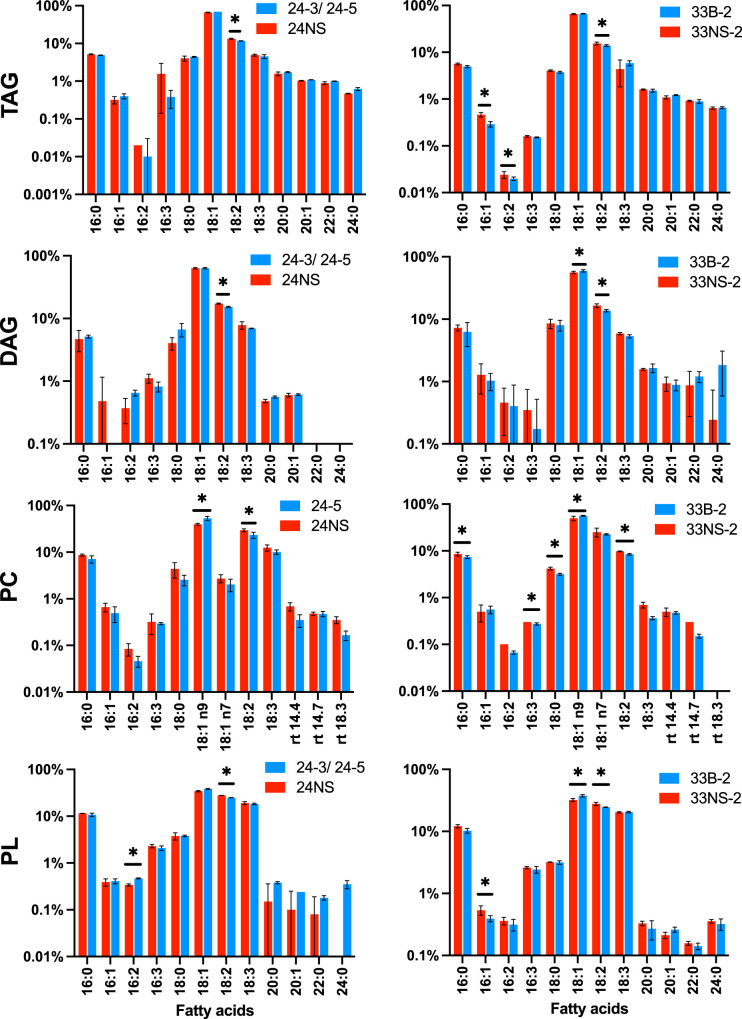
The fatty acid composition of endogenous lipids in 27DAF embryos is changed by PDAT overexpression. Fatty acids are abbreviated with carbon numbers and number of double bonds separated by a colon. The unsaturated fatty acids were oleic (18 : 1), linoleic (18 : 2) and alpha-linolenic (18 : 3). Results show means ± SE (*n* = 4). * indicates statistically significant changes (*P* < 0.05). Abbreviations: NS, null segregant; TAG, triacylglycerol; DAG, diacylglycerol; PC, phosphatidylcholine; PL, total polar lipids.

Because PDAT overexpression resulted in a consistent reduction in oil accumulation in different lines (Figure 1), we examined endogenous lipids in seeds at a time when lipid synthesis was high (27DAF). As anticipated, the overall seed composition in both NS controls and PDAT overexpressors was dominated by TAG (90%) and polar lipids (8.5%). However, when the fatty acid compositions of TAG, DAG and polar lipids were compared, notable differences were identified between NS controls and PDAT overexpressors (Figure 5). All three lipid classes showed significant decreases in their polyunsaturated fatty acid (PUFA) content (linoleic and alpha-linolenic acids) in PDAT overexpressing lines. The changes were larger in the polar lipid fraction, especially in PC, where a compensatory rise in oleate could be seen for the PDAT overexpressors.

More detailed analysis of the fatty acid content and species distribution in the TAG from dried seeds of the 33 line showed that the changes in degree of unsaturation were maintained at maturity. There was a small (−12%, *P* < 0.1), not significant, decrease in the amount of oleate per seed on PDAT overexpression, but a 25% (*P* < 0.0001) drop in 18 : 2 and a 30% drop (*P* < 0.0001) in 18 : 3, accounting for the apparent relative increase in oleate (Supplementary Spreadsheet S1). In terms of C54 TAG species, there was no significant change in the amount of C54 : 3 (−3%), but highly significant decreases in C54 : 4 (−19%; *P* < 0.005), C54 : 5 (−25%, *P* < 0.0001) and C54 : 6 (−32%, *P* < 0.00005). These decreases are seen to comparable degrees in each of the three 33 genotypes (Supplementary Figure S8).

Because of the alterations in the fatty acid distribution in polar lipids, we examined them further in 27DAF embryos. Data for the other main phosphoglycerides in two different PDAT overexpressing lines and their corresponding null segregants are shown in Supplementary Figure S6. Increases in the % oleate were seen for all four phosphoglycerides (phosphatidylethanolamine, phosphatidylglycerol, phosphatidylinositol and PC (shown in Figure 5)). In PC the main compensatory alteration was a decrease in linoleate and α-linolenate and an increase in the relative proportion of oleate in the PDAT overexpressing lines (Figure 5). A similar effect was seen for the other phosphoglycerides although, in general, it was less pronounced than for PC (Supplementary Figure S6). In contrast with the phosphoglycerides, the major chloroplast lipids, monogalactosyldiacylglycerol (MGDG) and digalactosyldiacylglycerol (DGDG) showed no consistent differences between PDAT overexpressors and their null-segregating controls (Supplementary Figure S6).

Taken together it appears that PDAT overexpression resulted in a shift in the composition of acyl chains available for phospholipid and TAG synthesis with little effect on galactolipids.

### Metabolic control analysis

**Figure 6.**
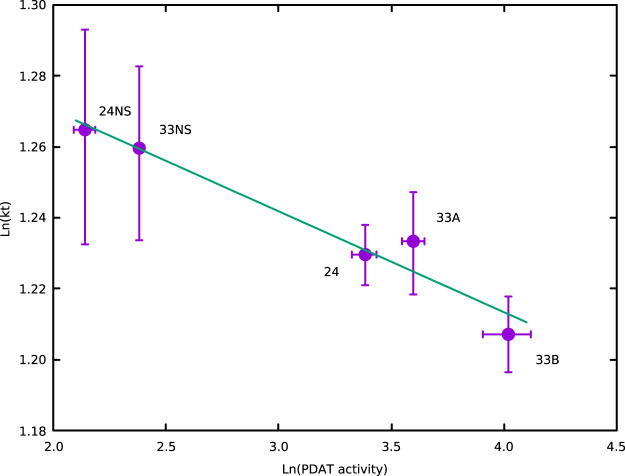
Flux control coefficient estimate for PDAT. The regression line has a slope highly significantly different from zero (*P* < 0.005) with a value of −0.028 ± 0.004(SE).

The values of the fluxes to TAG and the activities for PDAT in the non-segregant controls and the overexpressors for the three lines 24, 33A and 33B (Supplementary Table S1) approximated to a straight-line relationship on a log-log plot (Figure 6) with a negative slope to the regression line of −0.028 ± 0.004, corresponding to an estimate of the *in planta* flux control coefficient for PDAT in the non-segregants. Though small, the negative control coefficient is significantly different from zero (*P* < 0.005). The natural variation in PDAT activity between the non-segregants of lines 24 and 33 also seems consistent with this relationship. The three separate estimates using Eqn. (2) gave an average value of −0.027 (Supplementary Table S1), though this result is less precise as it does not use all the information in the data.

### The spatial distribution and relative amounts of TAG and PC molecular species differ in PDAT overexpressors

**Figure 7.**
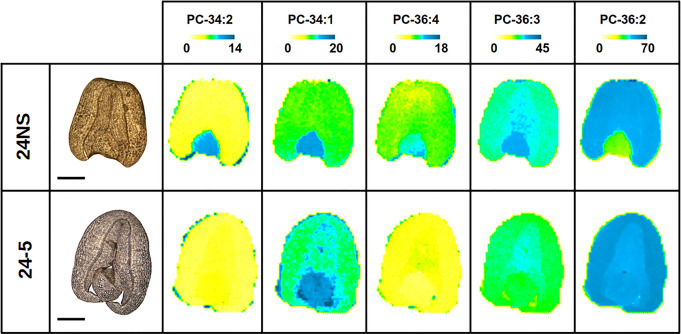
MALDI-MS imaging of selected PC molecular species in mature oilseed rape seeds for PDAT overexpressor (line 24-5) and null segregant control (24NS). Bright-field images are shown at the left of each row. Scale bar = 1 mm. The distribution of molecular species are shown with an adjusted mol% to show relative distributions. Above each image is a colour scale with yellow and blue representing low and high levels, respectively. Numbers at either end of the coloured bar represent the scale of that image. The molecular species are labelled with the total number of fatty acid carbon atoms and double bonds (e.g. PC-34 : 1 has 34 carbon atoms and one double bond).

**Figure 8.**
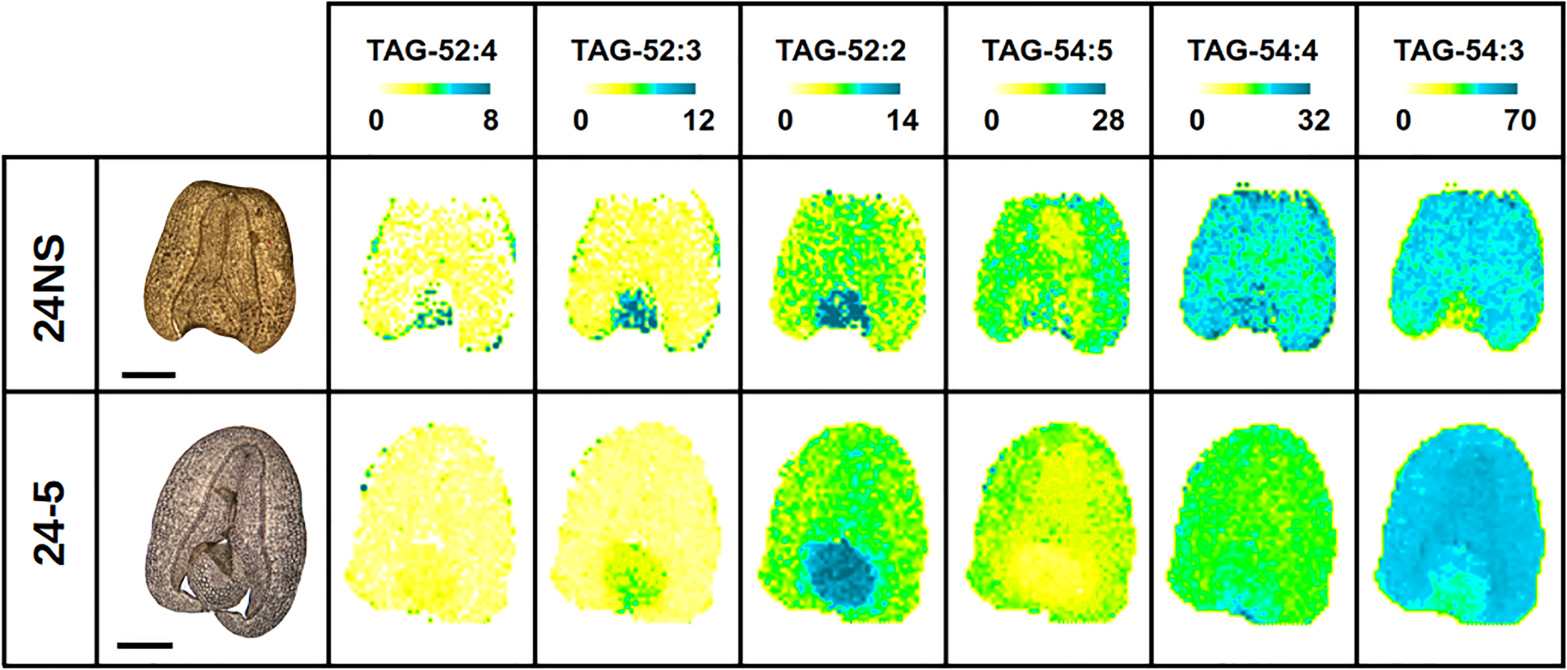
MALDI-MS of selected TAG molecular species in mature oilseed rape seeds for PDAT overexpressor (line 24-5) and null segregant control (24NS). Bright-field images are shown at the left of each row. Scale bar = 1 mm. The distributions of molecular species are with an adjusted mol% to show relative distributions. Above each image is a colour scale with yellow and blue representing low and high levels, respectively. Numbers at either end of the coloured bar represent the scale of that image. The molecular species are labelled with the total number of fatty acid carbon atoms and double bonds (e.g. TAG-52 : 4 has 52 carbon atoms and four double bonds).

Since our analysis of endogenous lipids showed differences for PDAT overexpressors (Figure 5 and Supplementary Figure S6), we expanded our analysis to include MALDI-MS imaging to visualise the spatial distributions of TAG and PC molecular species. Previous studies with *B. napus* [44] provided a useful context, and the spatial distribution of molecular species and the relative amounts of PC and TAG (Figures 7 and 8 and Supplementary Figure S7) were comparable between *B.napus* cv. Westar previously used and a similar low erucic acid rapeseed oil (LEAR) DH12075 background cultivar used here for production of PDAT transgenics.

A comparison of the mol% distribution of molecular species for PC (Table 1) and TAG (Table 2) showed differences for *B.napus* cv. DH12075 compared with PDAT overexpressing line 24. The differences are most obvious for the PC species where most (and all the predominant species) are significantly different.

**Table 1:** Distribution (mol%) of phosphatidylcholine molecular species is changed in PDAT overexpressors

Species	Null segregant (24NS)	PDAT overexpressor (24-5)
PC-34 : 3	0.9 ± 0.1	0.5 ± 0.1*
PC-34 : 2	3.9 ± 0.2	2.6 ± 0.3*
PC-34 : 1	10.5 ± 1.0	10.8 ± 0.2
PC-34 : 0	0.4 ± 0.3	0.4 ± 0.4
PC-36 : 6	0.1 ± tr.	tr.
PC-36 : 5	1.2 ± 0.3	0.5 ± tr.*
PC-36 : 4	8.9 ± 1.0	5.1 ± 0.4*
PC-36 : 3	27.8 ± 0.7	20.8 ± 0.8*
PC-36 : 2	45.9 ± 1.1	57.9 ± 0.4*
PC-36 : 1	0.4 ± 0.4	1.4 ± 0.8

Distributions were estimated from MALDI-MS analyses. Means ± SD (*n* = 3) are shown. tr. = trace (<0.05%). * is significantly different from null segregant control lines with *P* < 0.05.

**Table 2.** Distribution (mol%) of triacylglycerol molecular species in wild type and PDAT overexpressors

Species	Null segregant (24NS)	PDAT overexpressor (24-5)
TAG-50 : 2	0.1 ± tr.	n.d.
TAG-52 : 6	0.2 ± 0.2	n.d.
TAG-52 : 5	0.3 ± 0.3	0.1 ± 0.1
TAG-52 : 4	1.3 ± 0.9	0.9 ± 0.7
TAG-52 : 3	2.2 ± 0.8	1.6 ± 1.1
TAG-52 : 2	6.0 ± 0.4	6.7 ± 1.5
TAG-52 : 1	0.3 ± 0.3	0.3 ± 0.2
TAG-54 : 8	0.1 ± 0.2	n.d.
TAG-54 : 7	0.8 ± 0.7	0.3 ± 0.3
TAG-54 : 6	2.4 ± 1.5	1.5 ± 0.9
TAG-54 : 5	13.4 ± 1.5	11.9 ± 1.7
TAG-54 : 4	20.1 ± 2.1	17.1 ± 2.4
TAG-54 : 3	45.4 ± 7.2	52.2 ± 6.0
TAG-54 : 2	3.5 ± 1.9	2.7 ± 1.5
TAG-54 : 1	0.2 ± 0.2	0.1 ± 0.1
TAG-56 : 5	0.3 ± 0.3	0.2 ± 0.2
TAG-56 : 4	0.7 ± 0.5	0.6 ± 0.5
TAG-56 : 3	1.6 ± 0.5	2.4 ± 0.9
TAG-56 : 2	0.8 ± 0.5	1.2 ± 0.9

Molecular species distributions (% total) were estimated from MALDI-MS analyses. Means ± SD (*n* = 3) are shown. tr. = trace (<0.05%), n.d. = not detected.

In general, a decrease in unsaturation was observed for PDAT overexpressing seeds, which agrees with the analysis of PC in total seed extracts (Figure 5) where oleate was increased at the expense of linoleate and α-linolenate. For TAG (Table 2), similarly there was a general decrease in highly unsaturated molecular species although these trends were not statistically different.

When the spatial distribution of major PC species was examined, some clear differences were found for the PDAT overexpressors compared with controls (Figure 7). For wild-type, there was a relative enrichment in the embryonic axis for all PC molecular species, except for PC 36 : 2. For PC 36 : 2, its distribution in WT was markedly enriched in the cotyledons compared with the embryonic axis. Remarkably, these heterogenous distributions of PC species were mostly eliminated in PDAT overexpressing seeds, except for PC 34 : 1. All other PC species were mostly uniformly distributed throughout the seed in the PDAT transgenic. When examining these lipids from a spatial perspective, it seems that the PDAT effect on fatty acid composition of PC was most evident in the embryonic axis.

For TAG molecular species, TAG 52 : 2 and TAG 52 : 3 showed an obvious relative enrichment in the embryonic axis, while the major molecular species, TAG 54 : 3 (see Supplementary Figure S7B), was more enriched in the cotyledons of the null segregant control (Figure 8). In contrast, the PDAT overexpressors showed a more general distribution of 54 : 3 throughout the seed and with smaller amounts of the more unsaturated species of TAG (52 : 4, 52 : 3, 54 : 5, 54 : 4). These data agreed with the mol% distribution of TAG molecular species shown in Table 2, and were consistent with measurements anticipated by TAG quantified in developing seed total lipid extracts (Figure 5).

**Figure 9.**
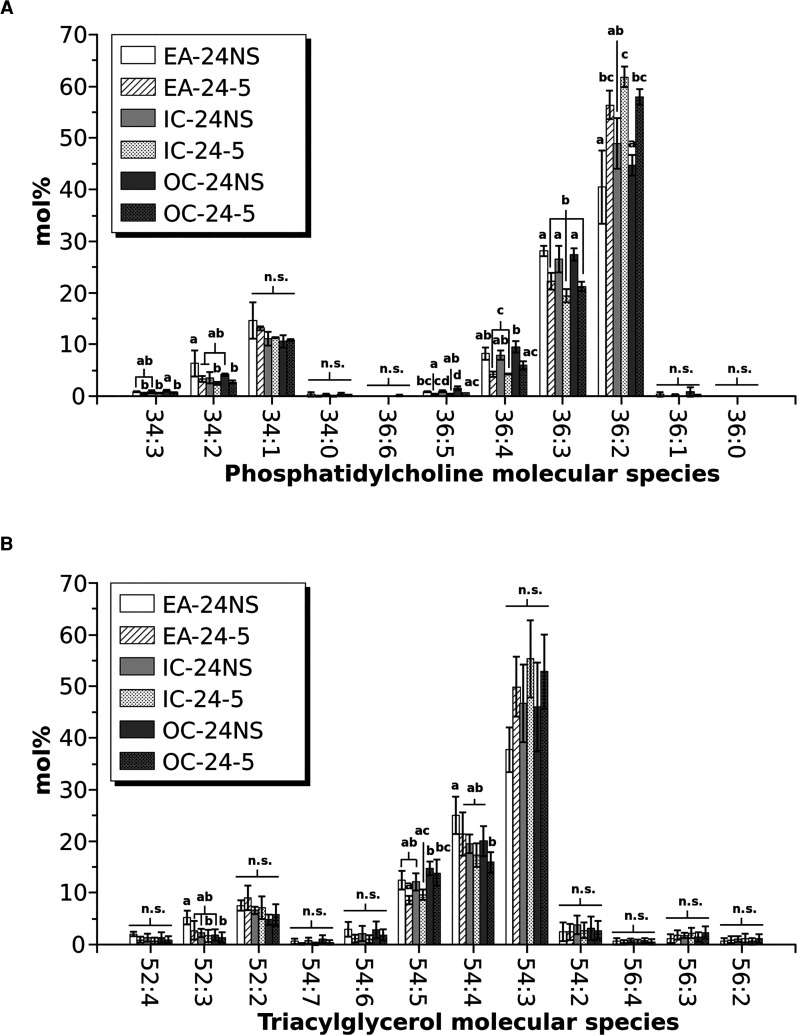
Percentage composition of molecular species in different parts of mature oilseed rape seeds. Distribution of (**A**) PC molecular species and (**B**) TAG molecular species in the embryonic axis (EA), inner (IC) and outer (OC) cotyledons for PDAT overexpressor (OE line 24-5) and null segregant control (24NS). Data show means ± SD (*n* = 3); statistics show one-way ANOVA; Tukey test, *P *< 0.05, with compact letter display; n.s. = not significant.

The visual distributions shown in Figures 7 and 8 were quantified in a relative manner in Figure 9 based on signal intensities summed separately for each seed part for both PC and TAG. For PC, the obvious increase in molecular species such as PC 36 : 2 were clearly observed in PDAT transgenics while the more unsaturated species (such as 36 : 3, 36 : 4, 34 : 2) were relatively reduced. Furthermore, a greater abundance of PC species such as PC 34 : 1 and PC 34 : 2 in the embryonic axis and a lower abundance of PC 36 : 2 for wild type was readily observed. As anticipated by visual inspections of the sections in Figure 7, these tissue-specific differences in PC compositions between the embryonic axis and cotyledonary tissues were markedly reduced in the PDAT transgenic seeds. The low variability (SD) among individual seed sections indicated that these relative differences in PC distributions were robust and reproducible from seed to seed.

Similar changes to the distribution of TAG molecular species were found among tissues and between the transgenic and non-transgenic seeds (Figure 9). The less unsaturated TAG 54 : 3 (nearly all triolein) tended to be higher in PDAT overexpressors, and more homogeneous between the embryonic axis and cotyledons compared with the non-transgenic background seeds. There were also lower amounts of the more unsaturated TAG species (52 : 3, 52 : 4, 54 : 4, 54 : 5, 54 : 6) in the PDAT overexpressing seeds. In all, PDAT overexpression not only resulted in a shift in unsaturated lipid composition, but also a marked disruption to the heterogeneous seed lipid distributions commonly observed in oilseeds.

## Discussion

### PDAT and flux to TAG

DGAT and PDAT are jointly responsible for the final steps in TAG formation in plant tissues. The relative contribution of these two enzymes varies considerably in different plants and plant parts [30]. As a generalisation, PDAT appears to be more important in seeds that accumulate highly unsaturated oils. Moreover, for the biosynthesis of TAG containing unusual fatty acids, PDAT activity is vital [56]. Thus, PDAT from castor bean (*Ricinus communis*) has proven useful in manipulating Arabidopsis to accumulate significant amounts of hydroxy-fatty acids, notably ricinoleic acid [36–38]. Recently, further improvements to oil yields as well as the percentage content of hydroxy-fatty acids have been made by including simultaneous overexpression of glycerol 3-phosphate and lysophosphatidate acyltransferases in the transgenic manipulation [39]. In leaf tissues of Arabidopsis, PDAT also has been shown to be involved (together with DGAT) in TAG synthesis [57,58]. This has led to demonstrations of its role in protecting against non-esterified fatty acid-induced cell death [57] and in basal thermotolerance [59].

For the LEAR (Canola) lines of *Brassica napus*, as in this study, which accumulate over 60% oleate in their oils [1], it might be expected that DGAT would be more important than PDAT for catalysing the final steps of TAG formation. Indeed, measurements *in vitro* of their activities agreed with this conclusion [22] and an examination of lipid molecular species in developing seeds also suggested that DGAT was more important than PDAT [4]. Nevertheless, PDAT might still contribute to overall TAG accumulation in Canola, albeit probably to a minor extent; hence, we studied the impact on TAG through PDAT overexpression. Opposite to expectations, we noted that expression of the Arabidopsis PDAT1 gene in *B. napus* cv DH12075 resulted in consistent decreases in TAG yields in three independent transgenic lines (Figure 1). These decreases in TAG ranged from 3% to 15% on a per seed basis and were observed despite increases in PDAT activity of up to 6-fold (Figure 2).

Previously, we had shown that flux control in *B. napus* cv Westar could be studied by using oleate addition to increase TAG biosynthesis and diazepam to inhibit TAG formation, respectively [22]. These reagents were used to compare null-segregants with PDAT overexpressors (Figures 3 and 4). The experimental data indicated that there was little difference observed when using [U-^14^C]glycerol to label total lipids. However, because [1-^14^C]acetate could not be used successfully in *B. napus* cv DH12075, we had to develop a different methodology to estimate flux control coefficients (see [26]).

Though small in magnitude, the negative control coefficient for PDAT is surprising and not simply explicable. It would suggest that flux through PDAT probably does not make a large contribution to TAG deposition in oilseed rape, but that does not account for why an increase in activity appears to inhibit net TAG deposition. One possibility might be impacts on the metabolite pool sizes. It is known from MCA theory [20] and experiments that overexpression of enzymes can have larger impacts on metabolite concentrations than fluxes; an example is the overexpression of phosphofructokinase in potato tubers, which causes significant alterations to the concentrations of glycolytic intermediates, with reductions upstream and increases downstream, but has no measurable effect on glycolytic flux [60]. If extra PDAT has changed the relative proportions of the various substrates and products for LPAAT and DGAT in a way that reduces their net activities, then this could have a negative effect on the main route of TAG accumulation. This suggestion depends on LPAAT and DGAT having positive control coefficients, so that the induced changes in activity translate into an effect on the TAG accumulation flux. We have demonstrated the small but significant flux control coefficient for LPAAT *in planta* [26] and overexpression of DGAT had a positive effect on TAG accumulation [21,23], implying that it too has a positive control coefficient.

### Compartmentation of PDAT and DGAT activities?

In relation to the above hypothesis that increased PDAT activity results in a change in metabolite pool sizes and a net reduction in TAG synthesis, the work of Bates et al. [61] is pertinent. Bates and co-workers first noted the use of different pools of DAG during TAG formation in 2009 and, since that time, a model for TAG synthesis has been developed to explain experimental data (see [11,38,62,63]) in several plant systems. Recently, observations in the model plant Arabidopsis show that DGAT1 produces TAG from a rapidly produced PC-derived DAG pool while PDAT1 utilises a different and larger bulk PC-derived DAG pool [64]. Our observation that the amount of C54 : 3 TAG shows a 3% reduction in the seeds of the PDAT overexpressors, whereas there is a 12% reduction in the oleate content of the TAG (Supplementary Spreadsheet S1), suggests that there is a largely unaffected pathway for C54 : 3 synthesis. However, statistically significant reductions in oleate-containing C54 : 4 and C54 : 5 led to a net loss of oleate from TAG as a whole, which is consistent with increased utilisation of the bulk PC-derived DAG pool in PDAT1 overexpressors.

### Effect of PDAT on TAG composition

When PDAT has been manipulated in developing plant seeds there has sometimes been an alteration in the balance of fatty acids accumulated. For example, for Arabidopsis overexpression of intrinsic PDAT gave no change in the fatty acid profile [27,65] while overexpression of a flax PDAT increased alpha-linolenate [31]. In contrast, intrinsic PDAT overexpression in *C. sativa* led to a relative increase in linoleate at the expense of alpha-linolenate (without affecting total lipid content) [33].

Because overexpression of Arabidopsis PDAT1 in *B. napus* had led to a small (but consistent) reduction in TAG yield (Figures 1, 6 and Supplementary Table S1), we then examined the fatty acid composition of lipids in developing embryos (Figure 5 and Supplementary Figure S6). Compared with null-segregant controls, TAG, DAG, PC and total polar lipids all showed decreases in linoleate and alpha-linolenate percentages (Figure 5) with relative increases in oleate which was statistically significant in PC and total polar lipids. Similar alterations in the fatty acid composition of other phosphoglycerides were seen (Supplementary Figure S6) although these tended to be less obvious than for PC (Figure 5). In contrast, there were no consistent changes for the two galactosylglycerides, MGDG and DGDG, suggesting that the acyl chains for their synthesis were not altered by PDAT overexpression, which might be anticipated since these galactolipids are assembled in the plastid and not in the ER.

To supplement our observations that PDAT overexpressing lines showed changes in endogenous lipids, we used MALDI-MS imaging of mature seeds to visualise spatial distributions of individual molecular species of TAG and PC. This technique had been used previously in *B. napus* in order to reveal spatial and temporal alterations during seed development [44]. Because of the intimate involvement of PC in oil accumulation [9,11,63] we examined this phosphoglyceride as well as TAG, as the main accumulating oil component. As expected, the main PC molecular species accumulating was PC 36 : 2 (Table 1, Figure 7; Supplementary Figure S7a) in both null-segregants and overexpressors. Figure 7 shows relative distributions of PC molecular species and the null segregants are similar to those reported for cv Westar at 27DAF [44]. Thus, for example, 34 : 2 was localised in the embryonic axis (EA), 36 : 3 was found in both cotyledons and the EA while 36 : 2 was concentrated in the cotyledons. In contrast, PDAT overexpression eliminated the clear enrichment of PC molecular species in the EA. Only for 34 : 1 was there a slightly higher relative concentration in the EA, with an increased concentration of 36 : 2 in the EA of the PDAT overexpressors relative to DH12075. Comparative data are also shown in Figure 9 where the results from several individual control or overexpressor replicates are presented. The data in Figure 8 for TAG molecular species also reveal differences in spatial distributions between a control and an overexpressor although these tended to be less obvious than for the PC molecular species.

The clear change in fatty acid distribution for both phosphoglycerides and accumulating non-polar lipids referred to earlier, is also obvious in the MALDI-MS images in Figures 7 and 8 and the tissue-localised distributions shown in Figure 9, as well as in total summed percentages shown in Tables 1 and 2. Thus, in Figure 7, the PC species 34 : 1 and 36 : 2 show an overall enrichment while 36 : 3 and 36 : 4 are reduced on PDAT overexpression. Similarly, in Figure 8, TAG 54 : 3 is enriched while 54 : 4 and 54 : 5 are reduced on PDAT overexpression. These data are supported by comparing total seed profiles in Tables 1 and 2, and emphasise that a major effect of PDAT overexpression is to reduce the relative amount of unsaturation as observed in the important intermediate PC as well as the final oil component TAG. Interestingly, a similar decrease in unsaturation was found when PDAT was overexpressed in *Camelina sativa* [33]. In that report it was suggested that an important reason for the alteration in fatty acids could be that channelling of fatty acids from PC into TAG (by increased PDAT activity) would prevent normal rates of desaturation. Our data, presented here, would agree with that conclusion. Indeed, if expression of Arabidopsis PDAT1 reflected only its substrate preference without other influencing factors then one would expect an increase in TAG unsaturation [27]. This was clearly not the case. The unexpected reduction in oil content in mature seeds found for *B. napus* (Figure 1) could be explained if increased PDAT activity pulled fatty acids away from the Kennedy pathway (i.e. DGAT-synthesised TAG) which is predominant in oilseed rape [4]. Indeed, independent confirmation of the relative importance of the Kennedy pathway (DGAT-synthesised TAG) compared with PDAT in *B. napus* can be attributed to gene expression measurements during seed development where DGAT rather than PDAT expression is notable when rapid oil accumulation takes place [66,67]. It is also noteworthy that, when PDAT was used to try to enhance accumulation of an unusual fatty acid (such as ricinoleic acid), its expression together with a delta12-hydroxylase led to a decrease in total seed oil. On the other hand, expression of both PDAT and DGAT (together with the hydroxylase) restored seed lipid levels [68]. Such data confirm the results of Marmon et al. [33] showing that the interactions of PDAT and DGAT are important in controlling both the quantitative and qualitative aspects of TAG biosynthesis in oil seeds.

### Conclusions

Overexpression of PDAT produced unexpected decreases in seed oil accumulation in a LEAR variety of *B. napus* (cv DH12075). In addition, a decrease in unsaturation of TAG and phosphoglycerides was observed. These alterations were confirmed by MALDI-MS where the normal distinct heterogeneous distributions of molecular species of PC and TAG were changed. Our data show that PDAT can have small but significant effects on lipid metabolism in *B. napus* which have implications for efforts to increase oil accumulation.

## Data Availability

Seed is available upon request from the corresponding author. Seed materials will be transferred under MTA. Additional experimental data is included in the Supplementary Files. MALDI-MS data, original data for Figures 5, 7, 8 and Supplementary Figure S8, and the Python code relating to Supplementary Table S1 can be downloaded from (https://mudshark.brookes.ac.uk/Projects/OilseedRape/PDAT/Data).

## Open Access

Open access for this article was enabled by the participation of Durham University in an all-inclusive *Read & Publish* agreement with Portland Press and the Biochemical Society under a transformative agreement with JISC.

## Abbreviations

DAF: days after flowering
DAG: diacylglycerol
DGAT: diacylglycerol acyltransferase
EA: embryonic axis
LEAR: low erucic acid oilseed rape
LPAAT: lysophosphatidate acyltransferase
MALDI: matrix-assisted laser/desorption ionisation
NS: null segregant
PC: phosphatidylcholine
PDAT: phospholipid: diacylglycerol acyltransferase
PG: phosphatidylglycerol
PUFA: polyunsaturated fatty acid
TAG: triacylglycerol
