# Supplementary material for: Overexpression of phospholipid: diacylglycerol acyltransferase in Brassica napus results in changes in lipid metabolism and oil accumulation
Source: Biochem J. 2022 Mar 31;479(6):805–23. doi: 10.1042/BCJ20220003 (PMC9022997; doi:10.1042/BCJ20220003)
Supplement: Supplementary Material [file BCJ-479-805-s1.pdf]

Supplementary data

Overexpression of phospholipid: diacylglycerol acyltransferase in *Brassica napus* results in changes in lipid metabolism and oil accumulation

Stepan Fenyk, Helen K. Woodfield, Trevor B. Romsdahl, Emma J. Wallington, Ruth E. Bates, David A. Fell, Kent D. Chapman, Tony Fawcett and John L. Harwood

Article reference: ……………………………………………

Contents

Supplementary figures S1-S8

Supplementary Table S1: Flux and enzyme activity results used in the calculation of the flux control coefficient of PDAT on TAG accumulation.

Additional Data

Additional data related to Figures 5, 7 and 8 of the main paper, and Supplementary Figure S8 is available at: [Projects/OilseedRape/PDAT/Data - Cell Systems Modelling Group (brookes.ac.uk)]

Supplementary Figures

Supplementary figure S1

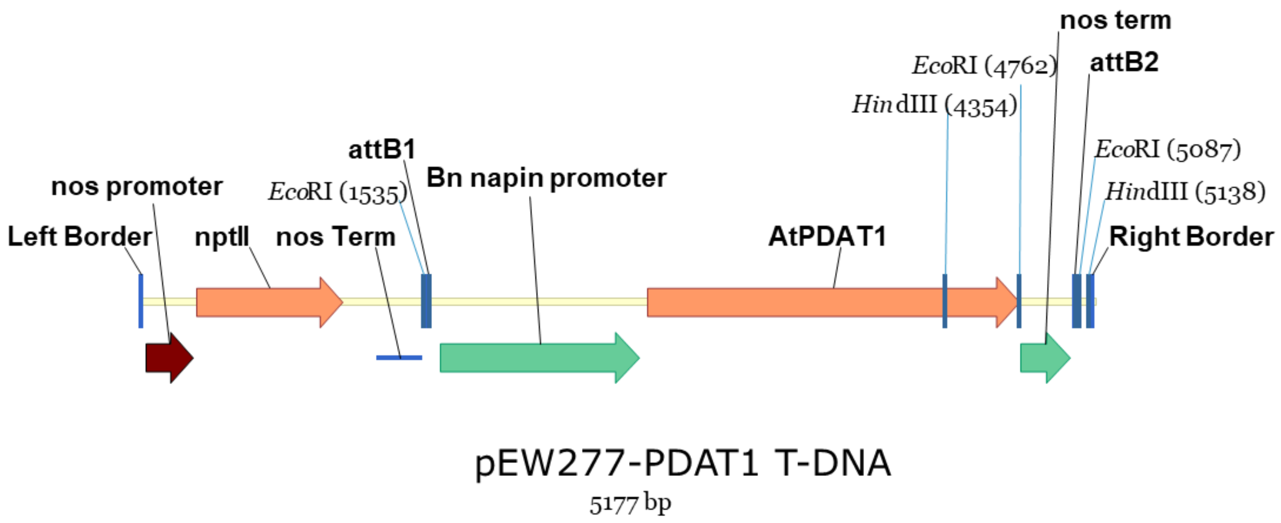

Supplementary figure S1. Schematic diagram of pEW277-PDAT1 T-DNA containing the *Arabidopsis thaliana* PDAT1 gene under the control of the *B. napus* napin promoter.

Supplementary figure S2.

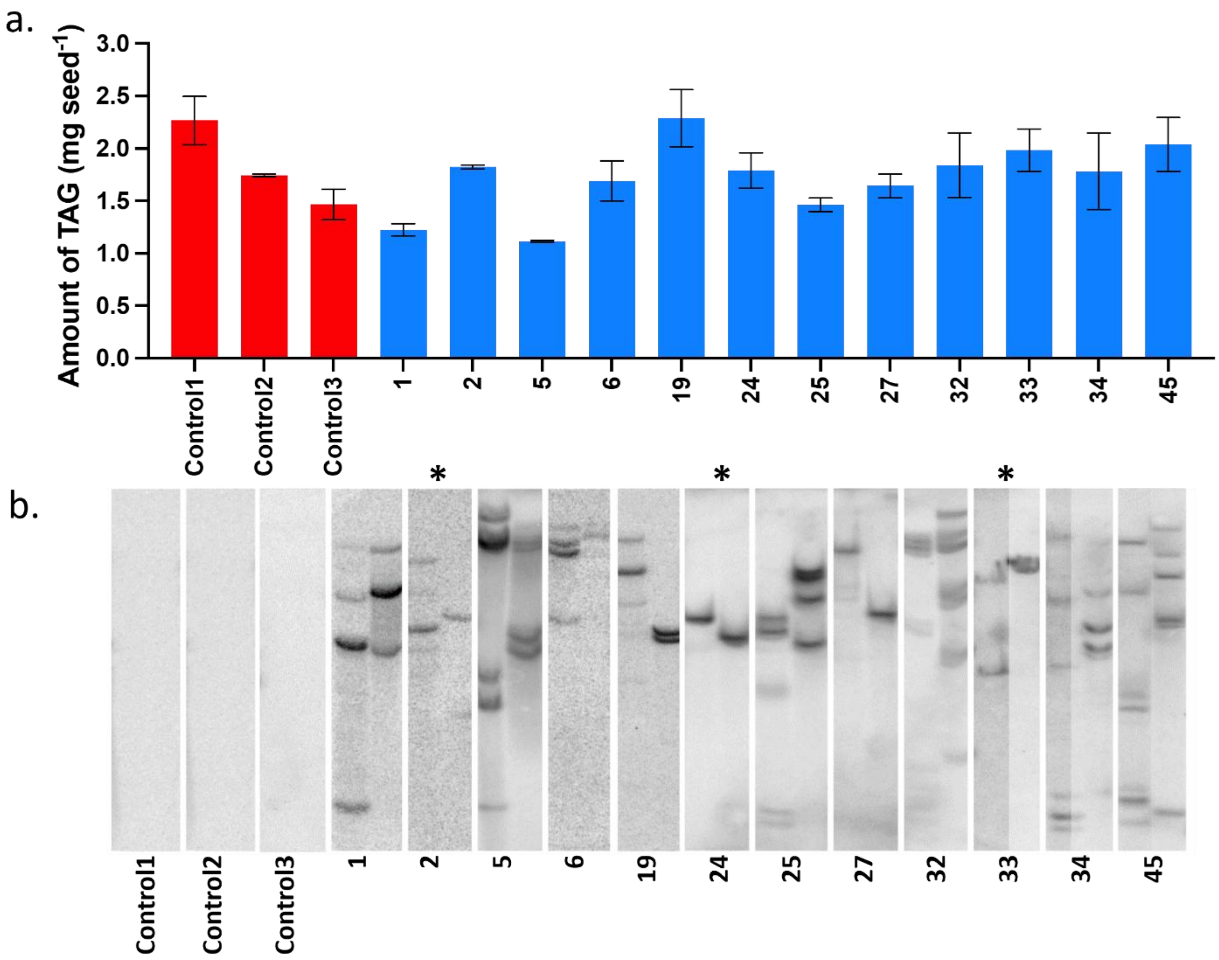

c.

Primer name	Sequence 5'-3'	Use	Reference
BN10-F	AGGAACTTGCATCCTCTTTGGA	qPCR copy number analysis	Woodfield et al., 2019
BN10-R	AATAACCGGTGGAGAATGTTCAG	qPCR copy number analysis	Woodfield et al., 2019
BN10-P	VIC-ATGCTGCTGGTGCTGTGGTT-TAMRA	qPCR copy number analysis	Woodfield et al., 2019
Npt2-F	CTCCTGCCGAGAAAGTATCCA	qPCR copy number analysis	Milner et al., 2018
Npt2-R	GCCGGATCAAGCGTATGC	qPCR copy number analysis	Milner et al., 2018
Npt2-P	FAM-TGGCTGATGCAATGCGGCG-TAMRA	qPCR copy number analysis	Milner et al., 2018
PDAT-F	TTGCCCCAGGATTCTTAGAC	PDAT1 presence PCR	This study
PDAT-R	ATGTACCCGGCACTTAGGAC	PDAT1 presence PCR	This study
AphIII-F	GGCAATGTGCCCTTATTCC	Plasmid backbone PCR	Woodfield et al., 2019
AphIII-R	GCATCAGGCTCTTTCACTCC	Plasmid backbone PCR	Woodfield et al., 2019

Supplementary figure S2. Selection of PDAT1 transgenic lines. (a) Amount of TAG per T1 seed in PDAT1 transgenic lines (blue bars) shown as ± S.D. of three independent biological replicates compared to three non-transformed controls (red bars). (b) Southern blot analysis of DNA from T0 plants probed with the npt11 gene. Each panel shows two lanes of DNA per plant digested with EcoR1 (left lane) or Hind111 (right lane) hybridised with the npt11 probe. * denotes lines taken forward to T1 generation. (c) Sequences of primers used to verify the presence of the transgene and copy number determination.

Supplementary figure S3.

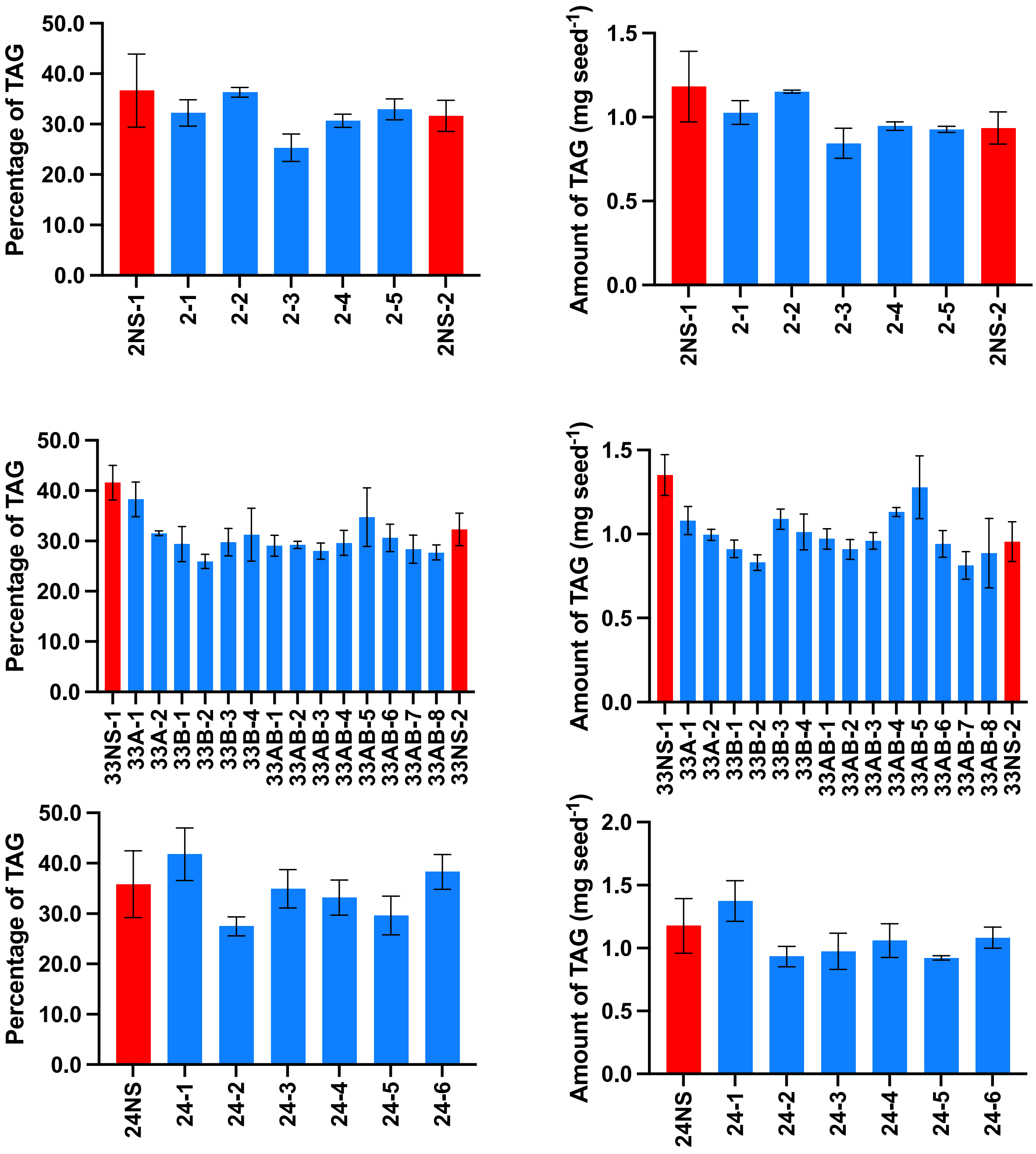

Supplementary figure S3. Amount and percentage of TAG in mature seeds of individual T_2 lines.

Supplementary figure S4.

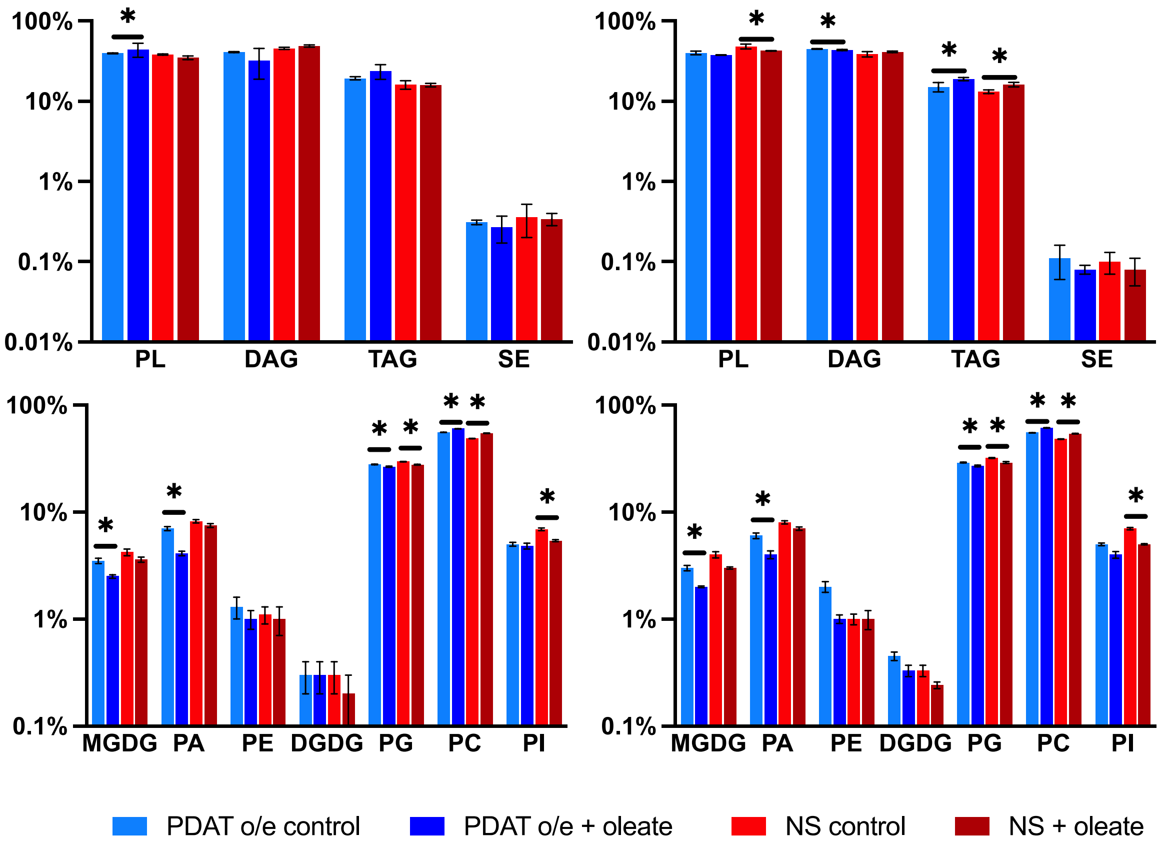

Supplementary figure S4. Exogenous oleate changes the distribution of radioactivity from [U-14C]glycerol between lipid classes. Left column—PDAT overexpressor line 33B-2, null segregant 33NS-2. Right column---PDAT overexpressor line 24-5, null segregant NS24 line. Results show means ± S.D. (n=3). * Significantly different from control ($p<0.05$). Lipid abbreviations: SE, sterol ester; MGDG, monogalactosyldiacylglycerol; PA, phosphatidic acid; PE, phosphatidylethanolamine; DGDG, digalactosyldiacylglycerol; PG, phosphatidylglycerol; PI, phosphatidylinositol.

Supplementary figure S5.

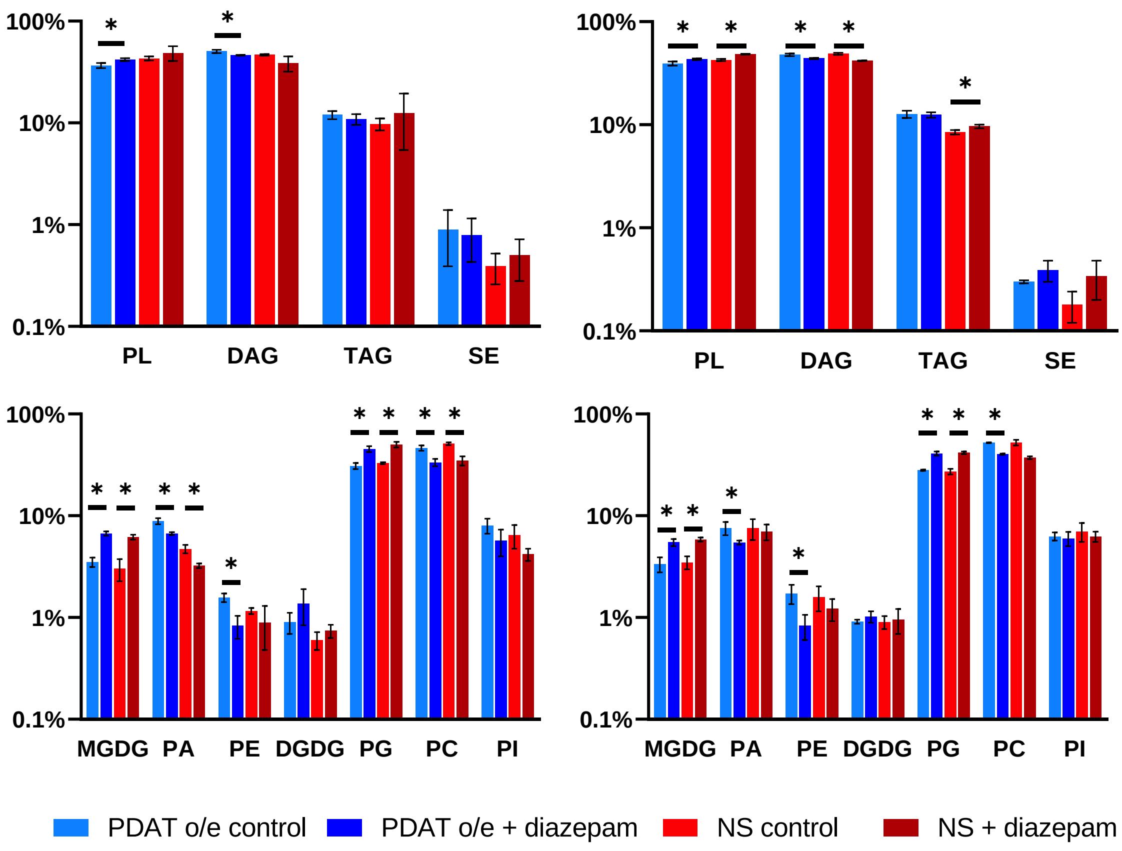

Supplementary figure S5. Diazepam alters the distribution of radioactivity from [U-14C]glycerol between lipid classes. Left column: PDAT overexpressor line 33B-2, null segregant line 33NS-2. Right column: PDAT overexpressor line 24-5, null segregant 24NS line. Results show means ± S.D. (n=3). * Significantly different from control ($p<0.05$). Lipid abbreviations as for Supplementary figure S4.

Supplementary figure S6.

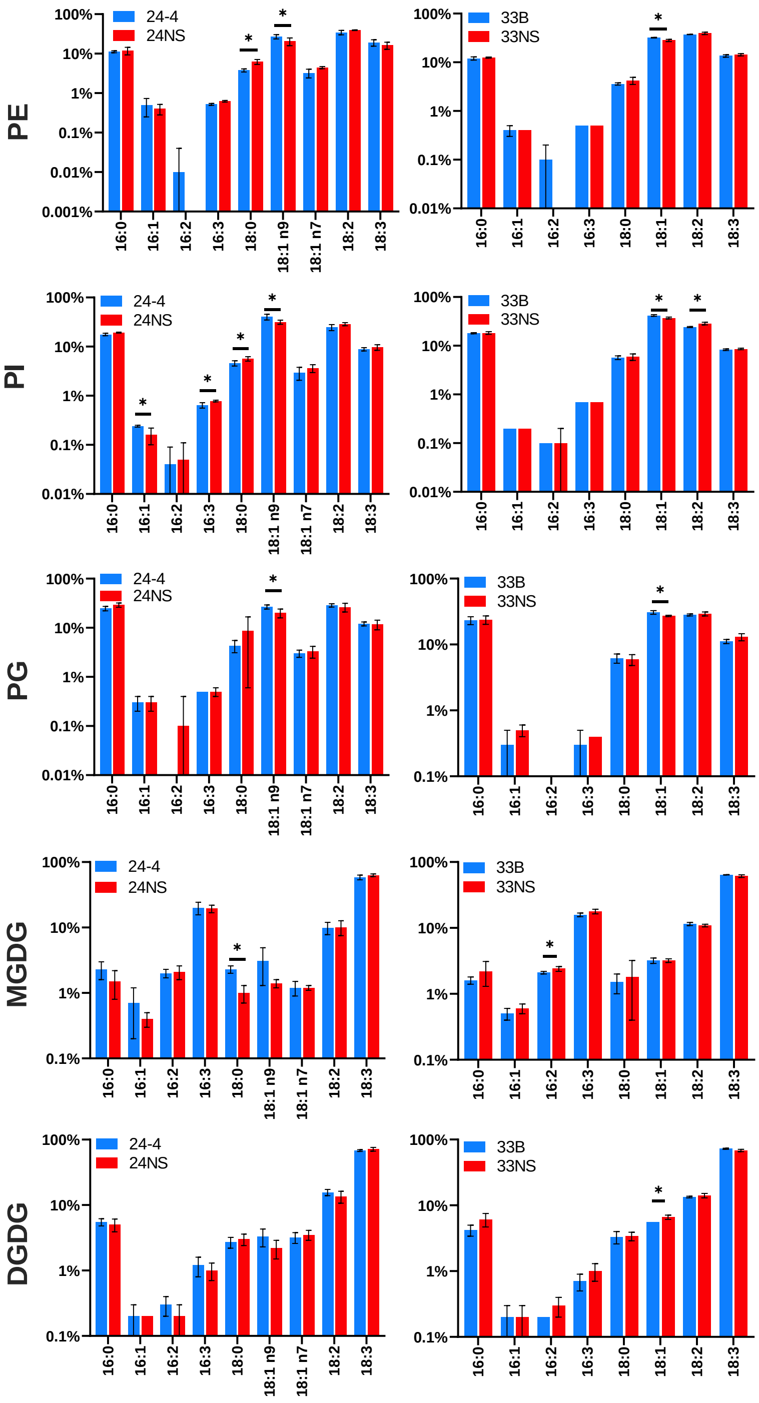

Supplementary figure S6. The fatty acid composition of individual polar lipids in 27 DAF embryos from null segregant (NS) controls and PDAT overexpressors (o/e). Data show means ± S.D. (n=3). * Significantly different ($p<0.05$) for o/e compared to NS controls. Lipid abbreviations as for Supplementary figure S4.

Supplementary figure S7.

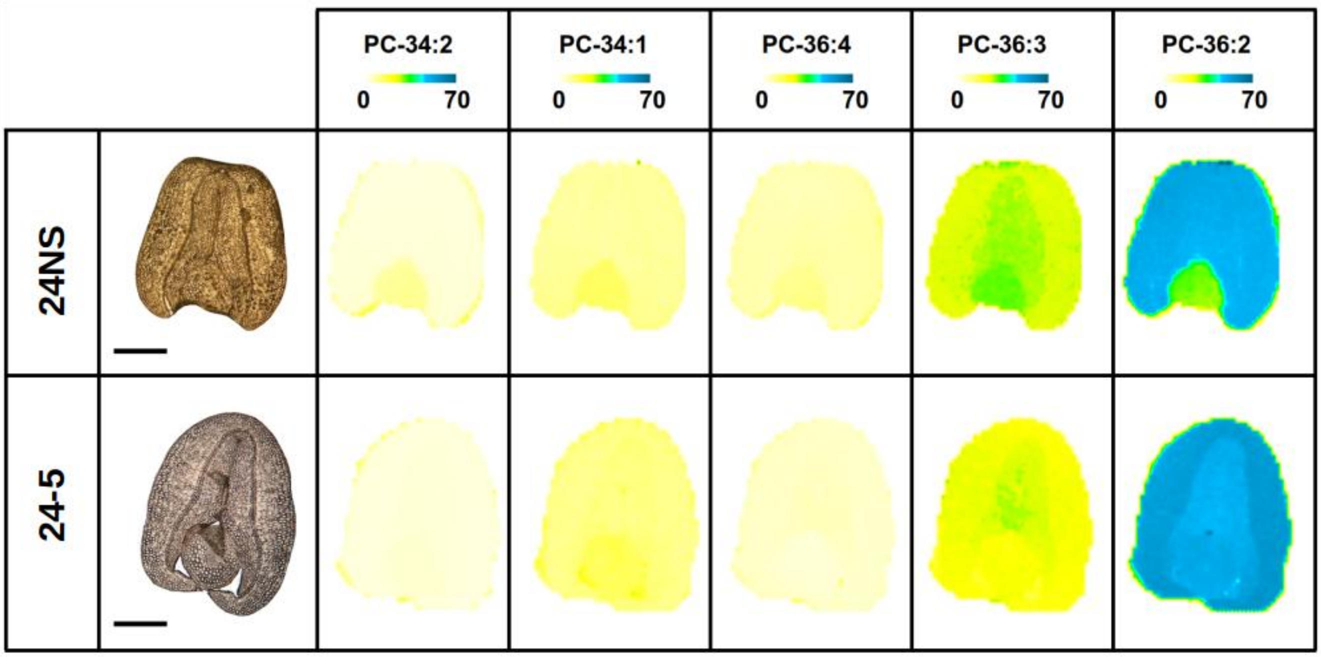

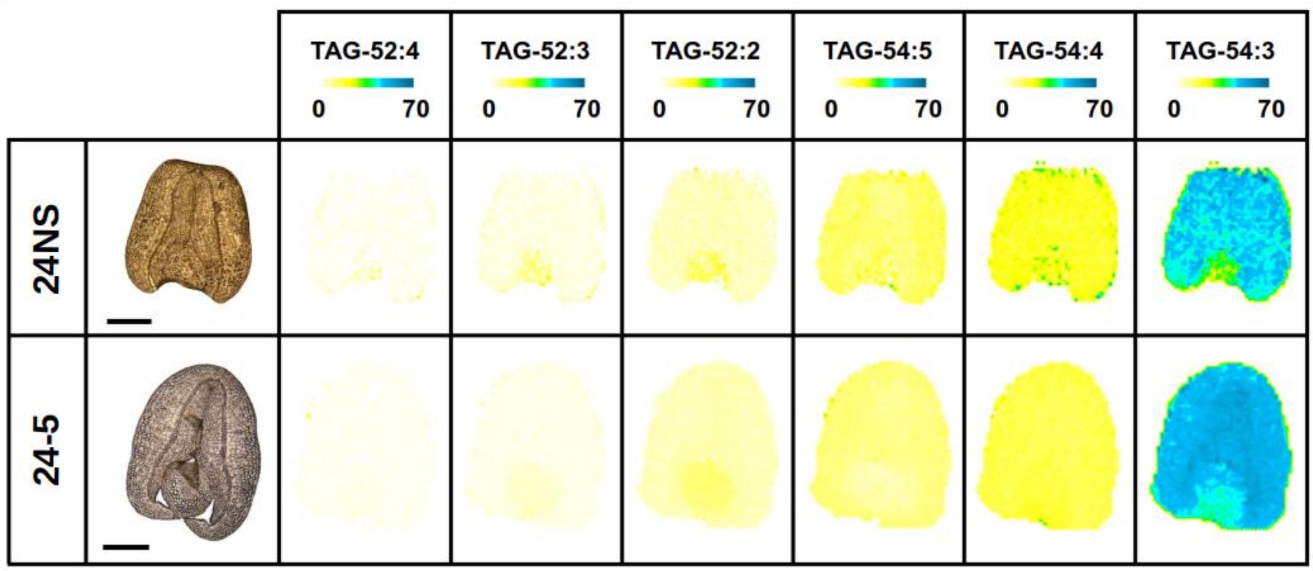

Supplementary figure S7. MALDI-MS imaging of selected PC (a) and TAG (b) molecular species in mature *B. napus* seeds for a PDAT overexpressor (line 24-5) and null segregant control (24NS). Details are as for Figs. 7 and 8 (main text) except that the distribution of molecular species is shown at a fixed mol% to show absolute distribution profiles.

Supplementary figure S8.

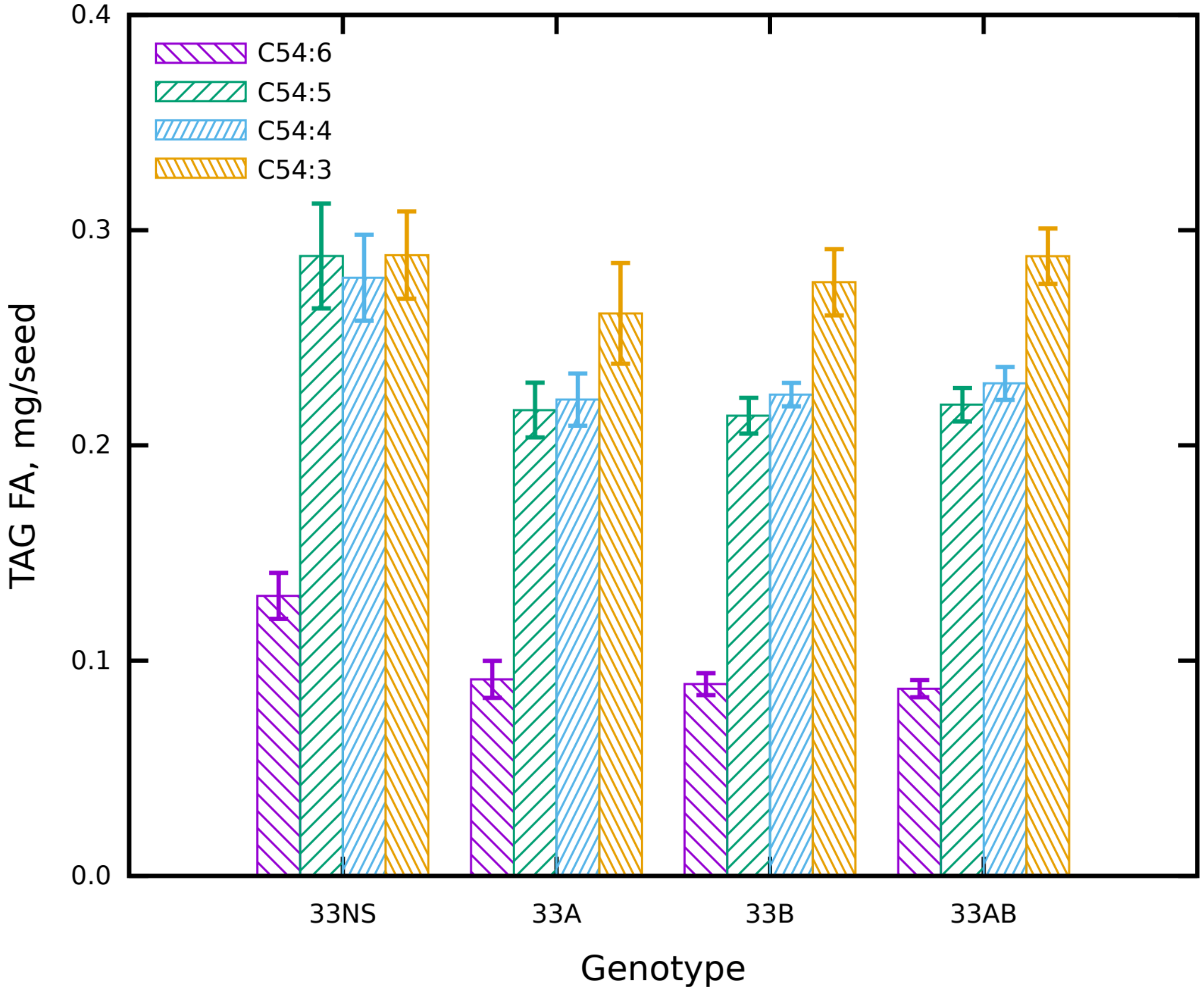

Supplementary figure S8. Changes in amounts of the major C54 TAG species per mature seed on PDAT over-expression. The three genotypes 33A, 33B and 33AB are compared with 33NS. In each cluster. The error bars show standard errors of the estimates. The n values are 33NS (6), 33A (6), 33B (12) and 33AB (24).

Supplementary Table S1. Flux and enzyme activity results used in the calculation of the flux control coefficient of PDAT on TAG accumulation.

	Plant Lines					
	**24NS**	**24**	**33NS**	**33A**	**33NS**	**33B**
**TAG, mg/seed**	1.175	1.053	1.153	1.040	1.153	0.964
**TAG, SE, mg**	0.125	0.052	0.099	0.030	0.099	0.034
**Kt**	3.542	3.433	3.524	3.420	3.524	3.344
**Relative flux change**	1.000	0.969	1.000	0.971	1.000	0.949
**PDAT**	8.510	36.538	10.840	29.420	10.840	55.540
**PDAT, SE**	0.408	1.873	0.240	1.620	0.240	5.950
**Relative enzyme**	1.000	4.294	1.000	2.714	1.000	5.124
**Flux Control Coefficient**	-0.022		-0.030		-0.032	
**SD**	0.023		0.025		0.016	

The initial embryo TAG content at the start of the exponential phase was taken as 0.034 ± 0.012 mg $seed^{-1}$ [26]. PDAT activity is expressed as TAG formed/mg protein/min. The errors on the flux control coefficients were estimated by Monte Carlo simulation as described in the Experimental section, taking into account all sources of variation.
